# Poor Physical Capacity Combined With High Body Fat Percentage as an Independent Risk Factor for Incident Hypertension in Chinese Suburb-Dwelling Older Adults

**DOI:** 10.3389/fpubh.2022.875041

**Published:** 2022-07-06

**Authors:** Peipei Han, Yuanyuan Zhang, Xiaoyu Chen, Zhenwen Liang, Xing Yu, Yuewen Liu, Sijia Sang, Jiayin Mao, Jingxuan Liu, Wuxiong Chen, Junxue Li, Yazhou Cheng, Yaqing Zheng, Ziwei Zhang, Ming Li, Qi Guo

**Affiliations:** ^1^Department of Rehabilitation Medicine, Shanghai University of Medicine and Health Sciences Affiliated Zhoupu Hospital, Shanghai, China; ^2^College of Rehabilitation Sciences, Shanghai University of Medicine and Health Sciences, Shanghai, China; ^3^Department of Rehabilitation Medicine, TEDA International Cardiovascular Hospital, Cardiovascular Clinical College of Tianjin Medical University, Tianjin, China; ^4^Department of Rehabilitation Medicine, Shanghai Health Rehabilitation Hospital, Shanghai, China; ^5^Fujian Provincial Hospital, Fuzhou, China

**Keywords:** older adults, physical capacity, body fat percentage (BF%), incident hypertension, Chinese

## Abstract

**Background:**

This study examined the effects of poor physical capacity and high body fat percentage (BF%) on the incidence of hypertension in Chinese suburb-dwelling older adults.

**Methods:**

This study was conducted on 368 Chinese suburb-dwelling participants aged ≥ 60 years without hypertension (mean age: 66.74 ± 5.59 years, 48.9% men). Poor physical capacity is defined by the Asian Working Group for Sarcopenia (AWGS) criteria as grip strength < 26 kg for men and < 18 kg for women or walking speed <0.8 m/s. High BF% was defined as values that are greater than the upper tertile for BF% as stratified by sex. The outcome was the incidence of hypertension.

**Results:**

Overall, 5.7% of subjects had both poor physical capacity and high BF%. After the average follow-up duration of 2 years, the incidence of hypertension was 39.7%, and those experiencing both poor physical capacity and high BF% had the highest incidence (81.0%). After multivariate adjustments, the incidence of hypertension was associated with the combination of poor physical capacity and high BF% [odds ratio (OR) = 6.43, 95% CI = 1.91–21.64] but not solely with poor physical capacity (OR = 1.11, 95% CI = 0.55–2.25) or only high BF% (OR = 1.37, 95% CI = 0.80–2.34).

**Conclusion:**

The combination of poor physical capacity and high BF% can significantly increase the incidence of hypertension in Chinese suburb-dwelling older adults. For hypertension prevention, ideally, we should strive toward decreasing body fat mass while simultaneously improving physical capacity.

## Introduction

As the aging population increases, hypertension has become one of the most prevalent diseases, affecting more than 70% of older people and contributing to the burden of cardiovascular disease, stroke, premature mortality, and disability (1–3). In addition, aging is also associated with a dramatic change in body composition and physical performance, such as an increase in the percentage of fat mass and a decrease in physical capacity, both of which are actively involved in metabolic regulation. Therefore, it is important to promptly and accurately identify and prevent these risk factors of hypertension in older adults.

Physical limitations can impair an independent lifestyle and quality of life. In fact, it is expected that the older population will experience declines in physical function and disability (4). Poor physical capability, as assessed by simple objective measures of muscle strength (grip strength) and physical performance (4-m walk test), has been shown to predict the onset of disability, loss of independence, and survival in older community-dwelling individuals (5). Several factors, such as endothelial dysfunction, oxidative stress, and inflammation, have been related to both arterial stiffness and muscular fitness (6–8). Muscle contraction-induced factors have an anti-inflammatory effect, but physical disability may cause a reduction in these factors, which in turn may increase the risk for cardiovascular disease, such as hypertension (9). Previous studies have emphasized the importance of preventing poor physical capacity when addressing hypertension, while other studies have suggested that poor physical capacity is not associated with hypertension (10). Given that the impact of poor physical capacity on hypertension is not fully understood, further research is needed to explore the association.

Aging is associated with significant changes in body composition. A recent large meta-analysis of nearly 200,000 individuals aged 65 and older showed a U-shaped relationship between body mass index (BMI) and mortality, with the lowest risk seen in those with a BMI between 24.0 and 30.0 kg/m (2). The risk began to increase when BMI exceeded 33 kg/m^2^ (11). A possible explanation involves the functions of adipose tissue. Adipose tissue produces leptin which may have protective effects on heart failure and decrease the risks of adiponectin in obesity. Similarly, in our previous study based on the Adult Physical Fitness and Health Cohort Study (APFHCS, ChiCTR 1900024880), we found that a high BMI is protective against sarcopenia (12). In contrast, some studies have shown a clear association between an increase in blood pressure (BP) and BMI. A systematic review and dose-response meta-analysis of more than 2.3 million participants found that the relative risk of hypertension was 1.49 for a five-unit increment in BMI (13). Because BMI and other abdominal obesity indicators may not adequately reflect the amount of body fat in some cases (14), we focused on the influence of high body fat percentage (BF%) on hypertension. A cross-sectional study found that dynapenia (grip strength) and abdominal obesity (waist circumference) were associated with a high prevalence of lipid and glucose metabolism disorders and metabolic syndrome (15). Therefore, it is necessary to investigate the effect of the combination of poor physical capacity and high BF% on the incidence of hypertension in Chinese older adults.

In this study, our purpose was to determine both the separate and combined effects of poor physical capacity and high BF% on the new-onset hypertension. We hypothesized that two pathological conditions would synergistically increase the risk of incident hypertension more than poor physical capacity alone or high BF% alone in Chinese suburb-dwelling older adults. This is a particularly significant study population, which is more likely to be healthy and have fewer activity limitations than those residing in care facilities (16). From a public health perspective, it is essential to identify hypertension-related risk factors to improve healthcare management and inform lifestyle intervention programs.

## Materials and Methods

### Study Participants

The APFHCS is a large prospective dynamic cohort study that mainly investigated the association between physical fitness and health status in a general adult population living in Tianjin, China. Participants were recruited for annual comprehensive health examinations and completed detailed questionnaires regarding their lifestyle and disease history. Our study population included 840 older individuals (age ≥ 60 years) from three areas of Tianjin, China, who joined the national free physical examination program from 2013 to 2014 at baseline. The inclusion criteria consisted of the following: (1) having undiagnosed hypertension; (2) having normal cognition (i.e., subjects who can communicate with interviewers or grant informed consent); and (3) subjects without disability or cardio-cerebrovascular diseases that affect the basic activities of daily living and geriatric assessments. A total of 453 subjects were excluded, i.e., 369 people with hypertension, 76 people with cardio-cerebrovascular diseases, three people with cancer, and five people who failed to undergo a physical examination. In total, 387 subjects enrolled in this study.

The cohort was invited to attend repeat questionnaire interviews and physical measurements after the 2 years from 2015 to 2016. During the follow-up duration, we excluded participants who died (*n* = 2), were bedridden (*n* = 2), and had those missing data (*n* = 15). Therefore, 368 participants were included. All participants provided informed consent prior to participation. The study was approved by the Ethics Committee of Tianjin Medical University.

### Definition of Hypertension

Blood pressure was measured two times from the upper left arm using a sphygmomanometer after 10 min of sitting, and the mean of these two measurements was taken as the BP value. Based on the eighth report of the Joint National Committee on Prevention, Detection, Evaluation, and Treatment of High Blood Pressure (JNC 8) (17), participants were defined as having hypertension with systolic blood pressure (SBP) ≥140 mmHg or diastolic blood pressure (DBP) ≥90 mmHg and/or the self-reported current treatment for hypertension with antihypertensive medication.

### Definition of Poor Physical Capacity and High BF%

All body composition indicators were measured using a direct segmental multifrequency bioelectrical impedance analysis (BIA) (InBody720; Biospace Co., Ltd, Seoul, Korea). According to the Asian Working Group for Sarcopenia (AWGS) criteria (18), poor physical capacity is defined as grip strength <26 kg for men and <18 kg for women or usual walking speed <0.8 m/s. Grip strength was measured using a dynamometer (GRIP-D; Takei Ltd, Niigata, Japan). Participants were asked to exert maximum effort two times using their dominant hand (19), and the result from the strongest hand was used for analysis. Usual walking speed (m/s) on a 4-meter course was used as an objective measure of physical performance (19).

Body fat percentage was assessed by the means of BF% (fat mass/weight). Because no consensus definition has yet been adopted (20), high BF% is defined by the highest tertile for BF%. The cutoff point by sex was ≥27.43% for men and was ≥37.00% for women.

### Assessment of Covariates

Data regarding sociodemographic variables, behavioral characteristics, and medical conditions were obtained *via* face-to-face questions. Sociodemographic variables included age, sex, marital status, educational level, and occupation. Marital status was classified as married (living together, divorced, separated, or widowed) or never married/single. Behavioral characteristics included smoking habits (current smoker or not), drinking habits (current drinker or not), and fall history. We have also described the methods of the International Physical Activity Questionnaire (IPAQ) and chronic conditions (such as diabetes, dyslipidemia, and osteoporosis). Details of the survey methods have been described in our previous study (12, 21).

### Statistical Analysis

All continuous variables with a normal distribution are expressed as the mean and standard deviation (SD), whereas data with an abnormal distribution are expressed as the median, with the 25–75% interquartile range given in parentheses. Categorical variables are expressed as percentages. Differences in the characteristics according to poor physical capacity and high BF% status were analyzed using analysis of variance (ANOVA), χ^2^ tests, and Kruskal-Wallis rank tests. Bonferroni corrected *p*-values (*k* = 6) were used for comparisons between poor physical capacity and high BF% categories. Logistic regression analysis was used to analyze the association between poor physical capacity and/or high BF% and incident hypertension. Crude was not adjusted. Model 1 was adjusted for age and sex. Model 2 was adjusted for Model 1 variables in addition to farming, illiteracy, widowed, living alone, smoking, drinking, appendicular skeletal muscle mass (ASM)/height^2^, IPAQ (binary categorical variables based on the median), fall history, diabetes, dyslipidemia, and osteoporosis. All statistical analyses were performed using SPSS version 25.0, and a *p*-value <0.05 was considered statistically significant.

## Results

### Characteristics of Subjects

Table 1 presents the baseline characteristics of all patients according to the incidence of hypertension during follow-up. The final study included 368 participants (48.9% men) with a mean age of 66.74 ± 5.59 years. According to the outcome of the average 2-year follow-up survey, 146 (39.7%) of the subjects (75 men and 71 women) with non-hypertension have developed hypertension. Subjects who had suffered from a hypertension event had a statistically significant higher BMI, BF%, and ASM/height^2^, and a lower walking speed than subjects without hypertension (*p* < 0.05). In addition, those with new-onset hypertension were more likely to be farming and had a higher prevalence of fall history and dyslipidemia (*p* < 0.05).

**Table 1:** Baseline characteristics of all patients following classification according to the incidence of hypertension during follow-up.

		**Hypertension event**		
**Characteristic**	**All** **(*n* = 368)**	**No event** **(*n* = 222)**	**Even** **(*n* = 146)**	***P*-value**
Age (y)	66.74 ± 5.59	66.34 ± 5.47	67.36 ± 5.75	0.088
Male (%)	180 (48.9)	105 (47.3)	75 (51.4)	0.255
BMI (kg/m^2^)	24.50 ± 3.32	23.84 ± 3.10	25.50 ± 3.39	<0.001
BF% (%)	29.71 ± 8.01	28.77 ± 8.14	31.14 ± 7.62	0.005
ASM/height^2^ (kg/m^2^)	7.04 ± 0.97	6.92 ± 0.98	7.21 ± 0.94	0.006
Grip strength (kg)	27.88 ± 9.05	27.83 ± 8.69	27.95 ± 9.60	0.895
Walking speed (m/s)	1.04 ± 0.17	1.06 ± 0.17	1.00 ± 0.17	0.002
IPAQ (Mets/wk)	2,110 (1,059–4,284)	2,099 (891–3,942)	2,184 (1,386–5,019)	0.289
Widowed (%)	37 (10.1)	24 (10.8)	13 (8.9)	0.599
Living alone (%)	41 (11.1)	22 (9.9)	19 (13.0)	0.398
Illiteracy (%)	97 (26.4)	57 (25.7)	40 (27.4)	0.718
Farming (%)	318 (86.4)	200 (90.1)	118 (80.8)	0.013
Smoking (%)	127 (34.5)	79 (35.6)	48 (32.9)	0.654
Drinking (%)	120 (32.6)	65 (29.3)	55 (47.7)	0.111
Fall history (%)	41 (11.1)	16 (7.2)	25 (17.1)	0.004
Diabetes (%)	33 (9.0)	15 (6.8)	18 (12.3)	0.092
Dyslipidemia (%)	147 (39.9)	79 (35.6)	68 (46.6)	0.039
Osteoporosis (%)	278 (75.5)	172 (77.5)	106 (72.6)	0.322

*The continuous variables are presented as mean ± standard deviation or median (25th−75th percentiles), the categorical variables are reported as percentages. ASM, appendicular skeletal muscle mass; BF%, body fat percentage; BMI, body mass index; IPAQ, international physical activity questionnaire; Mets/wk, metabolic equivalent task minutes per week*.

The baseline characteristics of subjects according to poor physical capacity and high BF% status are shown in Table 2. Overall, 102 (27.7%) subjects had only high BF%, 56 (15.2%) subjects had only poor physical capacity, and 21 (5.7%) subjects had both poor physical capacity and high BF%. Subjects who had suffered from poor physical capacity with high BF% or poor physical capacity alone were older (71.24 ± 5.97/69.04 ± 6.43 vs. 66.02 ± 5.10/65.89 ± 5.19, *p* < 0.001), had a weaker grip strength (17.72 ± 6.14/18.29 ± 5.67 vs. 30.37 ± 7.68/30.62 ± 8.44, *p* < 0.001), and a slower walking speed (0.86 ± 0.17/0.93 ± 0.17 vs. 1.07 ± 0.16/1.07 ± 0.15, *p* < 0.001). Those with poor physical capacity with a high BF% or high BF% alone had a higher BMI (27.29 ± 2.69/27.34 ± 2.67 vs. 23.29 ± 2.50/22.34 ± 2.93, *p* < 0.001) and BF% (38.87 ± 4.96/35.81 ± 5.75 vs. 26.05 ± 6.89/27.54 ± 6.73, *p* < 0.001) than other groups. Among these groups, the only poor physical capacity group had more female proportions (67.9 vs. 46.0%, Bonferroni *p* = 0.004), widowed (21.4 vs. 6.9%, Bonferroni *p* = 0.002), and had the lower ASM/height^2^ (6.45 ± 1.06 vs. 7.09 ± 0.93/7.31 ± 0.91, Bonferroni *p* < 0.001). The only high BF% group had a higher prevalence of dyslipidemia (52.0 vs. 34.4%, Bonferroni *p* = 0.004).

**Table 2:** Basic characteristics according to poor physical capacity and high BF% status.

**Characteristic**	**Normal (*n* = 189)**	**High BF% only** **(*n* = 102)**	**PPC only** **(*n* = 56)**	**PPC with high BF%** **(*n* = 21)**	***P*-value**
Age (y)	66.02 ± 5.10	65.89 ± 5.19	69.04 ± 6.43^a, b^	71.24 ± 5.97^a, b^	<0.001
Male (%)	102 (54.0)	54 (52.9)	18 (32.1)^a^	6 (28.6)	0.006
BMI (kg/m^2^)	23.29 ± 2.50	27.34 ± 2.67^a, c^	22.34 ± 2.93	27.29 ± 2.69^a, c^	<0.001
BF% (%)	26.05 ± 6.89	35.81 ± 5.75^a, c^	27.54 ± 6.73	38.87 ± 4.96^a, c^	<0.001
ASM/height^2^ (kg/m^2^)	7.09 ± 0.93	7.31 ± 0.91	6.45 ± 1.06^a, b^	6.79 ± 0.77	<0.001
Grip strength (kg)	30.37 ± 7.68	30.62 ± 8.44	18.29 ± 5.67^a, b^	17.72 ± 6.14^a, b^	<0.001
Walking speed (m/s)	1.07 ± 0.16	1.07 ± 0.15	0.93 ± 0.17^a, b^	0.86 ± 0.17^a, b^	<0.001
IPAQ (Mets/wk)	2,324 (1,386–5,040)	2,184 (1,021–5,371)	1,897 (953–3,287)	1,386 (297–2,772)	0.037
Widowed (%)	13 (6.9)	9 (8.8)	12 (21.4)^a^	3 (14.3)	0.017
Living alone (%)	14 (7.4)	12 (11.8)	10 (17.9)	5 (23.8)	0.029
Illiteracy (%)	51 (27.0)	20 (19.6)	18 (32.1)	8 (38.1)	0.180
Farming (%)	166 (87.8)	82 (80.4)	52 (92.9)	18 (85.7)	0.144
Smoking (%)	73 (38.6)	26 (25.5)	22 (39.3)	6 (28.6)	0.113
Drinking (%)	69 (36.5)	34 (33.3)	13 (23.2)	4 (19.0)	0.149
Fall history (%)	16 (8.5)	13 (12.7)	7 (12.5)	5 (23.8)	0.148
Diabetes (%)	14 (7.4)	9 (8.8)	6 (10.7)	4 (19.0)	0.293
Dyslipidemia (%)	65 (34.4)	53 (52.0)^a^	17 (30.4)	12 (57.1)	0.004
Osteoporosis (%)	146 (77.2)	72 (70.6)	44 (78.6)	16 (76.2)	0.586

*The continuous variables are presented as mean ± standard deviation or median (25th−75th percentiles), the categorical variables are reported as percentages. ASM, appendicular skeletal muscle mass; BF%, body fat percentage; BMI, body mass index; IPAQ, international physical activity questionnaire; Mets/wk, metabolic equivalent task minutes per week; PPC, poor physical capacity*.

*^a^P (bonferroni correction) < 0.05 vs. normal*.

*^b^P (bonferroni correction) < 0.05 vs. high BF% only*.

*^c^P (bonferroni correction) < 0.05 vs. PPC only*.

### Poor Physical Capacity and High BF% on Incident Hypertension

Table 3 presents the multivariable logistic regression analyses for the incidence of hypertension according to poor physical capacity and high BF% status. Compared to other groups, participants who had both poor physical capacity and high BF% had the highest incidence of hypertension. In the unadjusted model, the risk of the incidence of hypertension was progressively greater in the high BF% alone group [odds ratio (OR) = 1.67, 95% CI = 1.02–2.73] and the high BF% group than in the poor physical capacity group (OR = 8.30, 95% CI = 2.68–25.70). Adjusting for potential confounders of Model 2, high BF% when compared with poor physical capacity (OR = 6.43, 95% CI = 1.91–21.64) was still associated with a significantly higher risk for incident hypertension. However, this association was not observed in the high BF% alone group.

**Table 3:** Multivariable logistic regression analyses for the incidence of hypertension according to poor physical capacity and high BF% status.

**Status**	**Normal**	**High BF% only**	**PPC only**	**PPC with high BF%**	***P*-value**
Incidence of hypertension (%)	64 (33.9)	47 (46.1)	18 (32.1)	17 (81.0)^a, b, c^	<0.001
**Logistic regression analyses Odds Ratio (95% CI)**
Unadjusted	1.00 (Referent)	1.67 (1.02–2.73)^a^	0.93 (0.49–1.75)	8.30 (2.68–25.70)^a, b, c^	0.001
Adjusted Model 1	1.00 (Referent)	1.68 (1.03–2.76)^a^	0.91 (0.47–1.76)	7.96 (2.49–25.37)^a, b, c^	0.001
Adjusted Model 2	1.00 (Referent)	1.37 (0.80–2.34)	1.11 (0.55–2.25)	6.43 (1.91–21.64)^a, b, c^	0.023

*ASM, appendicular skeletal muscle mass; BF%, body fat percentage; CI, confidence interval; IPAQ, international physical activity questionnaire; PPC, poor physical capacity. Model 1 was adjusted for age and gender. Model 2 was adjusted for Model 1 variables in addition to farming, illiteracy, widowed, living alone, smoking, drinking, ASM/height^2^, IPAQ, fall history, diabetes, dyslipidemia and osteoporosis*.

*^a^P < 0.05 vs. normal*.

*^b^P < 0.05 vs. high BF% only*.

*^c^P < 0.05 vs. PPC only*.

## Discussion

In this study, we estimated the separate and combined effects of poor physical capacity and high BF% on new-onset hypertension in Chinese suburb-dwelling older adults. We found that the highest incidence of hypertension occurred among those with both poor physical capacity and high BF%. Furthermore, our results suggest that poor physical capacity alone or high BF% alone did not have a significant effect on the incidence of hypertension, while the combination of these two pathological conditions can independently predict the risk of developing hypertension after adjusting for potential confounders.

In our analysis, the incidence of hypertension was 39.7% after the average follow-up duration of 2 years, which is similar to a previous study (22). A large-scale longitudinal study had shown that 29.6% of suburb-dwelling older adults developed hypertension (23). The main possible reason for these differences is that the incidence of hypertension increases with age, and the average age of the above study (64.28 years) was lower than that of our study (66.74 years). Moreover, such variability to some extent can be attributed to differences in the characteristics, gender, and living conditions of the study sample.

Although there were significant differences in the grip strength (18.29 ± 5.67 vs. 30.37 ± 7.68, Bonferroni *p* < 0.001) and walking speed (0.93 ± 0.17 vs. 1.07 ± 0.16, Bonferroni *p* < 0.001) between the group of subjects who suffered from poor physical capacity alone and the group of normal individuals, our regression analyses revealed that poor physical capacity alone could not predict incident hypertension. A previous cross-sectional study supported our results, which suggested that hypertension is not associated with poor physical capacity (10). Furthermore, other researchers also found that a normotensive status rather than hypertension is associated with mobility limitations in those participants who aged ≥60 years (24). Indeed, recent reviews in the literature have described a possible association between high BP levels and poor physical capacity in older people (25, 26). This topic has been extensively discussed in the areas of geriatrics and gerontology, and a proposed theory is that a low and controlled hypertension phenotype can catalyze the development of frailty syndrome (25). The discrepancy between the results may be due to a difference in the sensitivity and specificity of the employed diagnostic tools and the characteristics of the participants. One major consideration is the age of the participants. Our study sample was approximately 10 years younger than those of previous longitudinal multicenter studies (27), which indicates that this condition may be time dependent. The other consideration is that poor physical capacity may increase the risk of incident hypertension due to the lack of physical activity. However, in our study, our population was relatively active and independent, and there were no significant differences in physical activity between the normal group and the poor physical capacity group [2,324 (1,386–5,040) vs. 1,897 (953–3,287), Bonferroni *p* > 0.05]. To date, studies on the causal relationships between poor physical capacity and incident hypertension are very few and limited, more future cohort studies are required to elucidate this relationship.

Obesity is increasing globally among Chinese older adults. Our results showed that high BF% alone can precipitate new-onset hypertension in a crude analysis but there was no difference after adjusting for potential confounders, which agrees with previous findings (28). On the one hand, the different outcomes may be due to the impact of the adjusted confounders, such as diabetes and dyslipidemia. Excessive intra-abdominal fat can be associated with type II diabetes, hypertension, dyslipidemia, coronary heart disease, and stroke. Despite having a normal BMI, those with an increased BF% are at increased risk of developing cardiometabolic diseases (29). On the other hand, because no consensus definition has yet been adopted, the cutoff values for BF% also have the potential to influence our results. Currently, the relationship between high BF% and hypertension has mostly been demonstrated based on cross-sectional studies, and more extensive longitudinal evidence is needed to clarify this relationship.

The present study results confirmed our primary hypothesis that the combination of poor physical capacity and high BF% can be an independent risk factor for incident hypertension. The results of our study show that the combination of poor physical capacity and high BF% had an incidence of hypertension of 81%, which was more than a 6-fold higher risk than those without poor physical capacity and high BF%. Although poor physical capacity alone or high BF% alone did not show significant effects on the morbidity of hypertension, their influence became obvious after superimposing. There are some reasons that may explain the joint effect of poor physical capacity and high BF% on hypertension. There was a clear negative relationship between fat mass and physical performance. Previous studies have shown a clear negative relationship between fat mass and physical performance. Poor physical capacity may cause physical inactivity, thereby leading to a reduction in energy expenditure, fat accumulation, and subsequent hypertension (30). Conversely, the activation of inflammatory pathways is mediated by adipose tissue. These inflammatory markers, such as tumor necrosis factor alpha (TNF-a), interleukin 6 (IL-6), C-reactive protein, and leptin, influence insulin resistance and growth hormone and cause an imbalance in protein synthesis, and lead to poor physical capacity (14). Furthermore, the additional weight resulting from body fat mass accumulation on the knee joints is associated with musculoskeletal impairments and physical disability. Therefore, it is possible to postulate that poor physical capacity and high BF% may potentiate each other to induce hypertension.

This study has a number of strengths. This was the first longitudinal prospective study to examine the separate and joint effects of poor physical capacity and high BF% on new-onset hypertension in Chinese suburb-dwelling older adults. In addition, it was also the first one to examine a uniquely defined group of suburb-dwelling older adults living in a discrete geographical area. Our participants were leading a more physically active lifestyle, which might differ from that of subjects in other geographical areas. Despite extensive efforts to curb the limits of our study, some limitations did exist. In our study, all participants in the present study were relatively healthy, as we did not include participants who were unable to participate in the free annual national physical examination, which inevitably led to selection bias. Due to this limitation, our results might in fact underestimate the prevalence of the combination of poor physical capacity and high BF% and its health impact. Despite this limitation, significant differences were still observed, implying that statistical power should not be a serious problem. In addition, the use of BIA to assess body composition presents a drawback, but it is well-correlated with magnetic resonance imaging (MRI) predictions and dual-energy X-ray absorptiometry (DXA). Furthermore, high BF% was identified as the highest tertile for BF% in our study, which is a lack of generally accepted reference values in the Chinese older adults. Despite controlling a considerable number of confounding factors, we cannot exclude all the possible confounders that may affect the results, such as endothelial dysfunction, oxidative stress, and inflammation, which correlate with BP. In the future, we will add relevant data to our subsequent study. In future research, we will enlarge the sample sizes and extend the follow-up years to determine the cut-off values for BF% and explore other lifestyle behaviors that might contribute to hypertension.

In our study, we found that poor physical capacity alone or high BF% alone did not have a significant effect on the incidence of hypertension, while the combination of poor physical capacity and high BF% may be an independent risk factor for incident hypertension in Chinese community-dwelling older adults. For older individuals with these two pathological conditions, ideally, we should pay attention to decreasing body fat mass while simultaneously improving physical capacity. Considering current findings, public health efforts should continue to promote regular physical activity and balanced nutrition to assist with the maintenance of optimal physical fitness.

## Data Availability Statement

The raw data supporting the conclusions of this article will be made available by the authors, without undue reservation.

## Ethics Statement

The studies involving human participants were reviewed and approved by Ethics Committee of Tianjin Medical University. The patients/participants provided their written informed consent to participate in this study.

## Author Contributions

PH wrote the main manuscript text. YZheng, ZZ, ML, and QG conceived and designed research. WC, JLi, and YC analyzed the data. XC discussed the results. ZL, XY, YL, SS, JM, and JLiu did the most research. YZhang made constructive comments during the review process. All authors contributed to the article and approved the submitted version.

## Funding

This work was supported by the National Natural Science Foundation of China (82172552), Shanghai Municipal Health Commission (20214Y0329), Shanghai Sailing Program (20YF1418200), and Scientific Research Foundation of SUMHS (SSF-21-03-007).

## Conflict of Interest

The authors declare that the research was conducted in the absence of any commercial or financial relationships that could be construed as a potential conflict of interest.

## Publisher's Note

All claims expressed in this article are solely those of the authors and do not necessarily represent those of their affiliated organizations, or those of the publisher, the editors and the reviewers. Any product that may be evaluated in this article, or claim that may be made by its manufacturer, is not guaranteed or endorsed by the publisher.
